# Use of Patient-Specific 3-Dimensional Printed Models for Planning a Valve-in-Valve Transcatheter Aortic Valve Replacement and Educating Health Personnel, Patients, and Families

**DOI:** 10.31486/toj.19.0106

**Published:** 2021

**Authors:** Jose D. Tafur Soto, Silvia Patricia Gironza Betancourt

**Affiliations:** ^1^ Department of Cardiology, Ochsner Clinic Foundation, New Orleans, LA; ^2^ The University of Queensland Faculty of Medicine, Ochsner Clinical School, New Orleans, LA; ^3^ Department of Internal Medicine, Universidad del Valle, Cali, Colombia

**Keywords:** *Aortic valve stenosis*, *imaging–three-dimensional*, *transcatheter aortic valve replacement*

## Abstract

**Background:** Aortic stenosis is a common disease of the elderly. Valve replacement with open surgery is the preferred therapy for many patients with low surgical risk. Bioprosthetic valve failure occurs in up to 66% of patients and has a worse prognosis when the mechanism of failure is stenosis compared to regurgitation.

**Case Report:** An 80-year-old female with a medical history of surgical aortic valve replacement, diabetes, chronic back pain, coronary artery disease, and hypertension was referred to the interventional cardiology clinic for heart failure symptoms. A bioprosthetic valve placement that was small for the patient's size (effective orifice area/body surface area 0.75 cm^2^/m^2^) resulted in symptomatic improvement that lasted for 7 years. The patient underwent an aortic valve-in-valve transcatheter valve replacement with excellent outcomes. Preoperative planning involved a patient-specific 3-dimensional printed patient model.

**Conclusion:** In patients at high surgical risk, transcatheter aortic valve replacement is a fundamental pillar of treatment. However, valve-in-valve procedures have specific anatomic challenges, such as the risk of coronary artery obstruction and the limitation of valve expansion inside a rigid bioprosthetic valve frame. In those difficult cases, interventional cardiologists must make precise decisions regarding the approach. Three-dimensional models can be printed with the patient's specific measurements. This approach represents truly personalized medicine and can serve as a tool for procedural planning, education of the health personnel involved in the case, and patient and family engagement.

## INTRODUCTION

Patient-specific 3-dimensional (3-D) printed models for structural heart disease have been demonstrated to be useful tools in interventional cardiology.^1,2^ These models have been used to improve outcomes in technically challenging cases, allowing for planning, education of the health team, and patient and family engagement. With the use of 3-D models, the heart team can visualize the patient's anatomy and any associated clinical problems and can provide the patient and family with a clear image of what is happening inside the heart and how the condition is going to be treated.^1,2^

Aortic stenosis is a common disease of the elderly; prevalence increases after 65 years of age, and after age 80 years, 1 of 3 people will have severe aortic stenosis.^3^ Surgical aortic valve replacement (SAVR) is a common treatment for severe aortic stenosis, but it is associated with the risk of patient-prosthesis mismatch (PPM), and the prevalence of severe PPM (effective orifice area [EOA]/body surface area [BSA] <0.65 cm^2^/m^2^) is 2% to 10%.^4^ Bioprosthetic valve failure occurs in up to 66% of patients^5^ and has a worse prognosis when the mechanism of failure is stenosis rather than regurgitation.^6^ PPM provides a partial explanation for this observation and is a problem especially in patients with small aortic roots who undergo SAVR. PPM occurs when the EOA of the implanted prosthetic valve is small related to the BSA. Patients with stenotic physiology who have bioprosthetic valve failure tend to have a combination of PPM and decreased leaflet mobility of the bioprosthetic valve. Walther et al showed a lower 5-year survival rate in patients with severe PPM vs patients without PPM (76.8% vs 81%) in an analysis of 4,131 patients.^7^ They also showed that an EOA/BSA <0.85 cm^2^/m^2^ was a significant risk factor for adverse cardiac events. Treatment options for PPM include reoperation^8^ and valve-in-valve (ViV) transcatheter aortic valve replacement (TAVR). Reoperation is associated with significant perioperative complications, including respiratory failure, reoperation for bleeding, acute myocardial infarction, need for intraaortic balloon pump, renal failure, sepsis or endocarditis, stroke, and gastrointestinal complications.^3,8^ Redo SAVR is a technically demanding procedure because of the scarred surgical field; the risk of iatrogenic injury to cardiovascular structures; and the higher risk of bleeding, transfusions, and transfusion-related morbidity compared to the first-time operation.^8^ ViV TAVR is a reasonable alternative but also has specific technical challenges. The rigidity of the bioprosthetic ring limits the size of the valve that can be used, further contributing to the PPM problem and causing a Russian doll–type effect (ie, the need to place a smaller prothesis every time to fit inside the preexisting one). This problem can be overcome by bioprosthetic valve fracture with high-pressure noncompliant balloon inflations; however, not all bioprostheses are susceptible to being fractured. Another challenge is that the risk of coronary artery occlusion is significantly higher with ViV procedures compared to TAVR in native aortic valves. The anatomic interaction between the valve components and the coronary ostia must be well studied to prevent such complications. In cases in which occlusion is predicted, preventive procedures such as stent snorkeling^9^ or BASILICA (bioprosthetic or native aortic scallop intentional laceration to prevent iatrogenic coronary artery obstruction)^10^ can be performed. A judicious study of the patient's anatomy is key to obtaining the best possible outcomes.

We present the case of a patient with challenging anatomy and the use of a 3-D printed computed tomography angiogram (CTA) model to help complete the procedure successfully.

## CASE REPORT

An 80-year-old female with a medical history of SAVR (19-mm St Jude Biocor Epic Supra with a true internal diameter of 16.5 mm), diabetes, chronic back pain, coronary artery disease, and hypertension was referred to the interventional cardiology clinic for heart failure symptoms. The patient had undergone successful valve placement 8 years prior with a bioprosthesis that was small for her size (initial EOA/BSA 0.75 cm^2^/m^2^). Despite the small bioprosthesis, the patient had symptomatic improvement that lasted for 7 years. One year prior to presentation, the patient developed dyspnea on exertion and angina.

On physical examination, she had normal heart rate, regular rhythm, and intact distal pulses. She had a harsh midsystolic murmur with a grade of 2/6 at the upper right sternal border radiating to the neck. The remainder of her examination was unremarkable. Her Society of Thoracic Surgeons (STS) estimated mortality risk was calculated at 7.14%.

Left heart catheterization confirmed elevated gradients across the bioprosthetic valve and showed nonobstructive coronary artery disease.

Her initial management was medical because of concern for performing a ViV procedure in a small bioprosthetic valve. However, after 4 weeks of medical therapy, the patient showed no clinical improvement, and her left ventricular function decreased from 65% to 30%.

**Figure 1.**
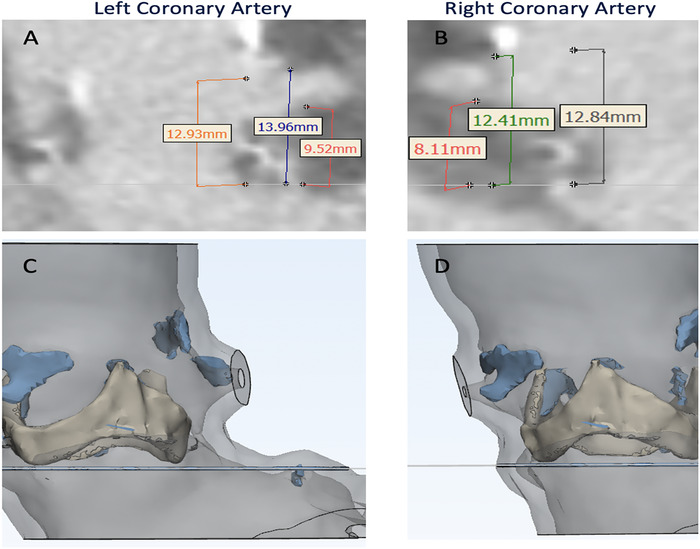
**Computed tomography angiography evaluation of coronary ostial height shows (A) the left coronary artery inferior height of 9.52 mm, superior of 13.96 mm, and frame of 12.93 mm, while (B) the right coronary artery has an inferior height of 8.11 mm, superior of 12.41 mm, and frame of 12.84 mm. Views C and D show 3-dimensional reconstructions of both coronary ostia.**

Echocardiogram showed an aortic valve area (AVA) of 0.64 cm^2^ (EOA/BSA 0.36 cm^2^/m^2^), mean aortic gradient of 46 mmHg, peak velocity of 4.7 m/s, and left ventricular ejection fraction of 30%. CTA showed that her aortoiliac system was appropriately sized for TAVR; bioprosthetic valve measurements in relationship to the aortic root complex are shown in Figure 1. The figure shows that the bioprosthetic valve is smaller than the patient's aortic root, a common finding in patients with PPM. Additionally, the images show the relationship of the bioprosthetic valve and the takeoff of the coronary arteries. A redo operation would have required resection of the bioprosthesis and a root enlargement procedure to be able to fit a bioprosthesis larger than the 19-mm Biocor valve that had been placed prior.

After evaluation by the heart team, the patient was considered to be high risk for surgery because of chronic back pain issues that would impede her recovery, the need for bioprosthetic valve resection and root enlargement, and frailty. The concern associated with ViV TAVR was possible coronary occlusion given the patient's low coronary takeoff and narrow sinuses of Valsalva. Even though the patient's bioprosthetic heart valve was considered low risk for coronary obstruction (leaflets were inside the frame), the plan to perform bioprosthetic valve fracture and overexpand the original frame was concerning for compromise of the coronary arteries.

**Figure 2.**
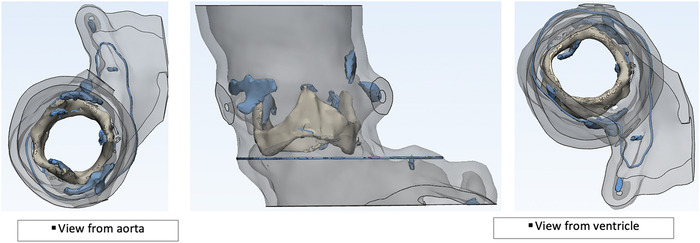
**Model of the aortic root using segmented blood volume.**

**Figure 3.**
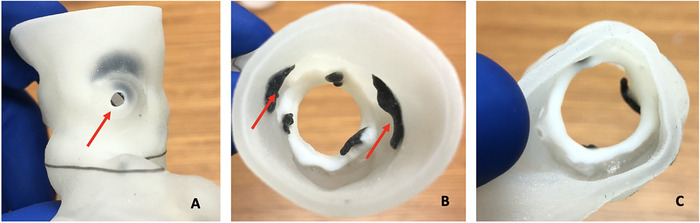
**In this 3-dimensional model of the aortic root—(A) left lateral view, (B) aortic view, and (C) ventricular view—the arrows show the coronary ostia.**

**Figure 4.**
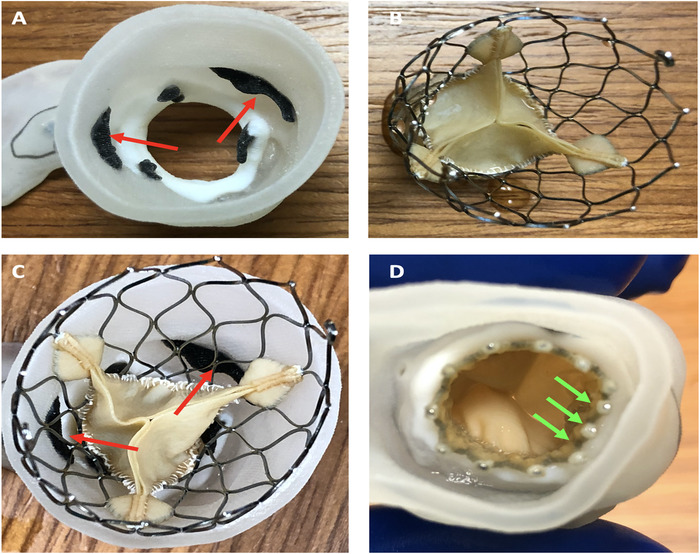
**View A shows the 3-dimensional model, and view B shows the 23-mm Evolut R valve (Medtronic). In view C, the arrows indicate the appropriate space for coronary perfusion and future cannulation. View D shows an inappropriately expanded Evolut R valve inside the bioprosthetic valve frame (arrows), confirming the need for bioprosthetic valve fracture.**

Segmented blood volume was used to create a 3-D model of the aortic root (Figure 2), and a 3-D printed CTA model was obtained (Figure 3). A 23-mm Evolut R valve (Medtronic) was placed inside the model to evaluate the anatomic interaction between the transcatheter valve and the aortic complex of the patient (Figure 4). Review of the model and the CTA showed that the bioprosthetic valve would need to be fractured to appropriately expand the transcatheter valve. After reviewing the CTA images and the 3-D model, the heart team felt that the sinuses of Valsalva had enough room for coronary perfusion despite expanding the bioprosthetic valve beyond its size. This expansion is achieved by inflating a noncompliant balloon 1 mm larger than the frame of the valve at high pressure (8 to 12 atmospheres), thereby causing a break in the valve frame and permitting the placement of a new transcatheter valve the same size or larger than the intact frame. This procedure is known as bioprosthetic valve fracture (Figure 4).

The model was used to explain the treatment strategy and the potential complications to the patient and her family. The model was also used to illustrate the specifics of the patient's anatomy to the treatment team, including cardiovascular fellows, nurses, and technologists.

The ViV TAVR procedure was performed successfully under conscious sedation. A 20-mm noncompliant balloon was inflated at 10 atmospheres to fracture the 19-mm bioprosthetic valve. A 23-mm Evolut R valve was placed using fluoroscopic landmarks of the bioprosthetic valve. At the end of the procedure, the coronary arteries remained patent; patency was confirmed with contrast injection. No complications occurred during or after the intervention. The patient was discharged home the next day in good condition.

When the patient was evaluated in the clinic 30 days after the procedure, she reported complete resolution of her symptoms. Two-dimensional echocardiography showed left ventricular ejection fraction of 40%, AVA of 2.38 cm^2^, mean aortic gradient of 7 mmHg, and EOA/BSA of 1.35cm^2^/m^2^, all of which were significantly better than her hemodynamic measurements after SAVR.

## DISCUSSION

PPM is a frequent problem after SAVR. Even though symptoms may initially improve, the longevity of such valves tends to be shorter compared to valves without PPM. When symptoms appear, treatment options are limited.^3^ Redo SAVR can be considered but often cannot be performed because of patient comorbidities.^8^ ViV TAVR has become one of the treatments of choice given its low invasiveness and satisfactory clinical outcomes. The PARTNER (Placement of Aortic Transcatheter Valves) 2 ViV Trial showed favorable survival, better hemodynamic status, better New York Heart Association class, and restored quality of life compared to redo SAVR.^11^ Those results have also been shown in other clinical trials.^12,13^

The necessary resources must be available to ensure that the geometry and positioning of the prosthetic valve and the patient's anatomy do not result in complications. Our patient had narrow sinuses of Valsalva and low takeoff of the coronary arteries, representing a high risk of coronary artery obstruction, especially with overexpansion of the bioprosthetic valve via bioprosthetic valve fracture. Having a specific 3-D model from her cardiac CTA and performing bench tests were very helpful and contributed to procedural success. For interventional cardiology, 3-D models offer unique opportunities to tailor surgical procedures to patients through the creation of physical models of patient anatomy derived from routine preprocedural imaging studies.^14,15^ These models not only visualize the anatomy but also how the patient's anatomy will behave during the cardiovascular intervention.^16^ An additional advantage of 3-D models is the opportunity to give information to patients and family members. Verbal explanations of the illness, complications, and procedures are often not enough; visual aids improve patients’ understanding of their clinical status and are thus a valuable tool for patient-physician interaction.

## CONCLUSION

PPM contributes to bioprosthetic valve deterioration. Reoperation is a treatment option but has major obstacles. ViV TAVR has demonstrated good hemodynamic and quality-of-life outcomes but also has problems, such as the risk of coronary artery occlusion and valve underexpansion because of rigid small bioprosthetic frames. Three-D models with the patient's specific anatomic characteristics are a useful tool for procedural planning and anticipating complications, as well as for family and healthcare team engagement.

## Notes

This article meets the Accreditation Council for Graduate Medical Education and the American Board of Medical Specialties Maintenance of Certification competencies for Patient Care and Medical Knowledge.
